# Effects of Tungsten Addition on the Microstructure and Corrosion Resistance of Fe-3.5B Alloy in Liquid Zinc

**DOI:** 10.3390/ma10040399

**Published:** 2017-04-10

**Authors:** Xin Liu, Mengmeng Wang, Fucheng Yin, Xuemei Ouyang, Zhi Li

**Affiliations:** 1 School of Material Science and Engineering, Xiangtan University, Xiangtan 411105, China; liuxin11509@smail.xtu.edu.cn (X.L.); xuemeiouyang89@smail.xtu.edu.cn (X.O.); lizhiclsj@xtu.edu.cn (Z.L.); 2 Key Laboratory of Materials Design and Preparation Technology of Hunan Province, Xiangtan University, Xiangtan 411105, China; 3 School of Material Science and Engineering, Shanghai Jiaotong University, Shanghai 200240, China; mengmengwang@sjtu.edu.cn

**Keywords:** Fe-B alloy, corrosion resistance, reticular boride, tungsten, molten zinc

## Abstract

The effects of tungsten addition on the microstructure and corrosion resistance of Fe-3.5B alloys in a liquid zinc bath at 520 °C were investigated by means of scanning electron microscopy, X-ray diffraction and electron probe micro-analysis. The microstructure evolution in different alloys is analyzed and discussed using an extrapolated Fe-B-W ternary phase diagram. Experimental results show that there are three kinds of borides, the reticular (Fe, W)_2_B, the rod-like (Fe, W)_3_B and flower-like FeWB. The addition of tungsten can refine the microstructure and improve the stability of the reticular borides. Besides, it is beneficial to the formation of the metastable (Fe, W)_3_B phase. The resultant Fe-3.5B-11W (wt %) alloy possesses excellent corrosion resistance to liquid zinc. When tungsten content exceeds 11 wt %, the formed flower-like FeWB phase destroys the integrity of the reticular borides and results in the deterioration of the corrosion resistance. Also, the corrosion failure resulting from the spalling of borides due to the initiation of micro-cracks in the grain boundary of borides is discussed in this paper.

## 1. Introduction

Because of the superior atmospheric corrosion resistance of zinc coating, and the excellent strength and formability of galvanized sheet steels, hot-dip galvanizing has been used as the most efficient and economical method to prevent steels from corrosion [1,2,3]. However, in a continuous galvanizing line (CGL), the immersed equipment (e.g., bearings, sink roll, stabilizer, corrector rolls, heaters etc.) are subjected to serious corrosion by molten zinc [4,5,6]. In such circumstances, finding materials that have a long service life even when they come into contact with liquid zinc flowing at elevated temperatures of 450 °C to 600 °C becomes rather challenging. A number of materials such as Stellite alloys (Co-Cr-W-C alloys), ceramics, spray coatings and other bulk materials have been developed to prolong the service life of the immersed equipment [7,8,9]; however, most of them cannot meet the rigorous working conditions in a CGL.

In recent years, much attention has been paid towards the casting of Fe-B alloys. The microstructures of the Fe-B alloys are comprised of the corrosion-resistant Fe_2_B phase and a ductile α-Fe matrix. This kind of alloy shows higher hardness and good corrosion resistance in molten zinc [10]. The reticular Fe_2_B phase acts as a physical barrier at the corrosion interface, and hinders the Fe-Zn reaction. Ma et al. [11,12,13,14] indicated that a Fe-B alloy containing 3.5 wt % B had good corrosion-resistance in molten zinc. However, when the liquid zinc bath temperature reached 520 °C, the corrosion of the Fe-3.5B alloy became severe because of the fracture and spalling of the reticular Fe_2_B phase at the corrosion interface [14,15]. Recently, Ma et al. [16,17] reported a new kind of Fe-B cast alloy with high chromium and nickel contents which can improve the corrosion resistance of the alloy even at 520 °C. However, according to their reports, cracks initiated from the reticular borides and caused the borides to spall off at the corrosion interface after only two days of immersion in the molten zinc.

It is well known that refractory metals such as molybdenum and tungsten show excellent corrosion resistance against molten zinc at high temperatures [18]. The excellent corrosion resistance of these alloys is mostly due to the high melting point and weak chemical affinity of such refractory materials with zinc. Xing et al. [19] investigated the effects of tungsten on the microstructure and fracture toughness of the Fe_2_B phase in a Fe-B-C cast alloy. The results showed that the Fe_2_B still displayed a reticular morphology and the fracture toughness was greatly increased with tungsten addition. A reticular morphology and high fracture toughness are important for excellent corrosion resistance in molten zinc. Inspired by Xing’s work, the effects of tungsten on the microstructure and the corrosion resistance in molten Zn bath of a Fe-3.5B alloy have been investigated in the current work, with the goal of improving the corrosion-resistance of the Fe-3.5B alloy by means of alloying. As mentioned above, the corrosion-resistance of Fe-B alloy is reduced sharply when the temperature of the zinc bath reaches 520 °C, so the test temperature is chosen as 520 °C in this paper.

## 2. Experimental

### 2.1. Preparation of Samples

The as-cast low carbon Fe-3.5B alloy samples containing different amounts of tungsten were prepared using a WK-I non-consumable vacuum arc furnace. The raw materials were pure iron (of 99.9 wt % purity), pure tungsten (of 99.9 wt % purity), and a Fe-B master alloy with 17 wt % B. Carbon and silicon were minor impurities in the Fe-B master alloy. The nominal composition of the samples is shown in Table 1. In order to ensure the chemical homogeneity of the alloys, each sample (with total weight of 25 g) was melted at least four times in a water-cooled copper mould under an argon atmosphere. After melting, ingots of dimensions 15 mm × 9 mm × 4 mm were cut from the alloys using a wire electric discharge machine.

### 2.2. Immersion Test

**Figure 1:**
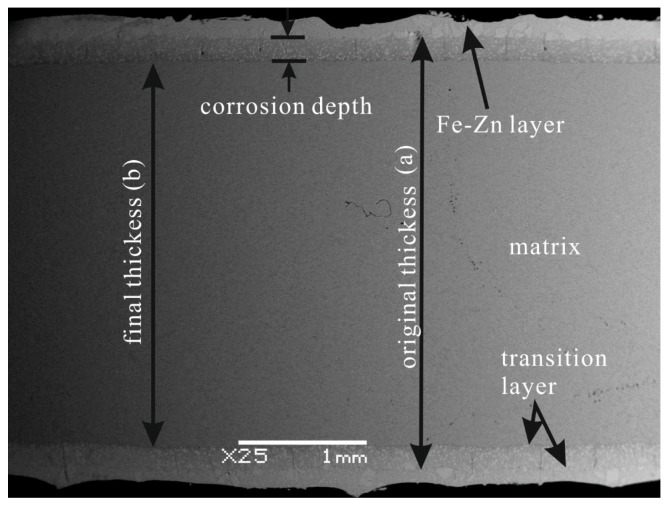
The schematic of specimen thickness measurement.

The as-cast Fe-3.5B alloys undergo uniform corrosion in liquid zinc, therefore, the corrosion depth method was utilized to analyze the corrosion resistance of the Fe-B alloys [20]. The static corrosion tests in molten zinc (99.9 wt % purity) were conducted in a graphite crucible placed in the vertical electric resistance furnace. The temperature was monitored by a thermocouple inserted in a quartz tube immersed in the molten zinc. The samples were mechanically polished with emery paper until 2000 grit. The initial thickness of the tested samples was obtained from the average value of 10 measurements of the distance between the two sides of the samples, measured accurately at multiple locations using a micrometer. Before the corrosion test, the samples tied with a tungsten wire (99.99 wt % purity) were ultrasonically cleaned in pure alcohol, and then immersed in the zinc bath at temperatures of 520 ± 5 °C for different time periods (24, 48, 72, 96 and 120 h). After the corrosion test the samples were withdrawn from liquid zinc, quenched in water and then embedded in resin. The after-immersion sample thickness was measured 10 times at 0.5 mm intervals from the center to both sides using the Smile-View software provided by the SEM manufacturer. The decrement in the average thickness was used to calculate the corrosion rate, using the equation given below [11]:

*R* = (*a − b*)/2*t* (1)

where *R* is the corrosion rate (mm·h^−1^), *a* is the original thickness (mm) of each sample before the immersion test, *b* is the final thickness of the matrix after the corrosion test (mm), and *t* is the corrosion time (h). The factor of 1/2 in the equation is used to reflect the average thickness change in the sample. Figure 1 depicts the schematic of the thickness measurement.

### 2.3. Material Characterization

A 4% HNO_3_ etching solution was used to reveal the as-cast and corrosion interface microstructure details. Further metallographic examination and compositional analyses of the various phases in the samples were performed using a JSM-7600F and a JSM-6360LV scanning electron microscope (SEM, JEOL, Tokyo, Japan) equipped with an energy dispersive X-ray spectrometer (EDS, Oxford Inca, Oxford Instruments, Oxfordshire, UK), a JXA-8230 electron probe microanalyzer (EPMA, JEOL, Tokyo, Japan) along with a wavelength dispersive X-ray spectrometer (WDX). The elemental distribution was investigated in detail using linear and mapping analysis by the EPMA. The constituent phases in the alloys were determined from the X-ray diffraction patterns (XRD, Rigaku-IV, Rigaku Corporation, Tokyo, Japan) generated by a D8 advance diffractometer operating at 40 kV and 40 mA with Cu Kα radiation.

## 3. Results

### 3.1. As-Cast Microstructure and Phase Identification

**Figure 2:**
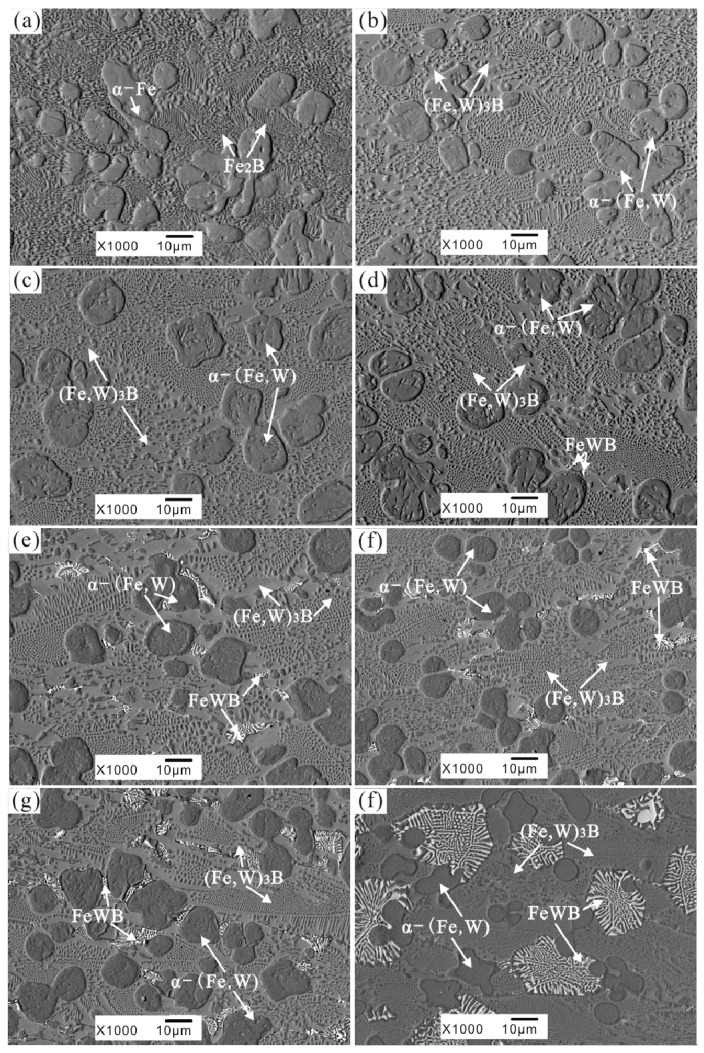
The as-cast microstructures of Fe-3.5B alloys containing different amounts of tungsten (**a**) A0-0 wt % W (**b**) A1-2 wt % W (**c**) A2-4 wt % W (**d**) A3-6 wt % W (**e**) A4-8 wt % W (**f**) A5-11 wt % W (**g**) A6-13 wt % W (**h**) A7-15 wt % W.

The as-cast microstructures of the test alloys are shown in Figure 2. The microstructure of A0 alloy (Figure 2a) represents a typical hypoeutectic morphology with a primary α-Fe solid solution and reticular Fe_2_B borides. In A1–A7 as-cast alloy, the hypoeutectic morphologies can still be kept (Figure 2b–h), but the reticular borides are refined greatly with the addition of W (Figure 2e). When tungsten (W) content reaches 6 wt % (A3 alloy), white boride particles precipitated at the edge of the grey borides (Figure 2d). As the W addition exceeds 8 wt %, the morphology of the white borides has a tendency to transform into flowerlike shapes as shown in Figure 2e–h. In the A7 alloy the formation of the big flowerlike white borides destroys the integrity of the reticular borides, which are important for the corrosion resistance of the Fe-B alloy.

**Figure 3:**
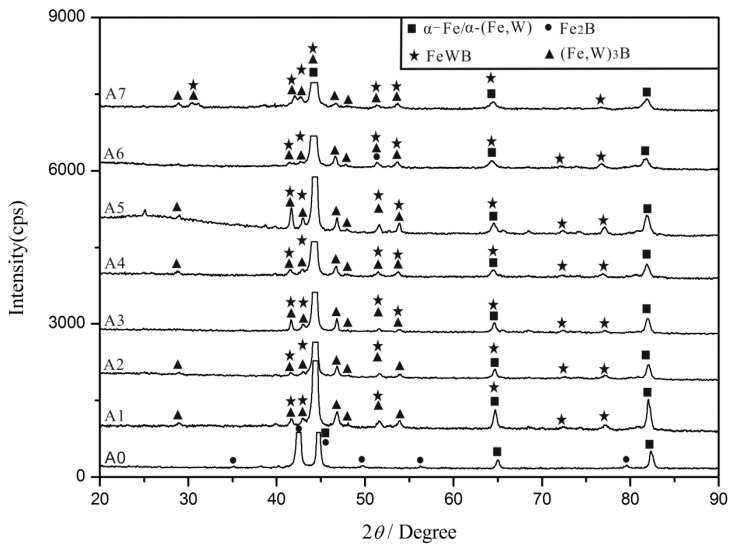
X-ray diffraction patterns of Fe-3.5B alloys containing different amounts of tungsten.

WDX and XRD analyses were carried out to further reveal the composition and the structure of the cast alloys with different W contents, the experimental results are shown in Table 2 and Figure 3. The metal matrix is identified as the α-Fe solid solution (JCPDS 06-0696), and the eutectic borides mainly comprise of the M_2_B-type and M_3_B-type (M = Fe, W) borides, such as Fe_2_B (JCPDS 36-1332) and Fe_3_B (JCPDS 39-1316). The white phase is identified as FeWB (JCPDS 23-0307) ternary boride. In Figure 3, the XRD peaks of α-Fe in the Fe-3.5B-*x*W alloys shift to the left compared to the A0 alloy. The reason behind this shift is the large difference in the atomic radius of the Fe atom (*r* = 1.26 Å, *a* = *b* = *c* = 2.886 Å) and the W atom (*r* = 2.02 Å, *a* = *b* = *c* = 4.060 Å). The solution of W atom with bigger atomic radius in α-Fe enlarges the lattice parameters.

### 3.2. Corrosion Rate and Morphology of the Corrosion Layer

**Figure 4:**
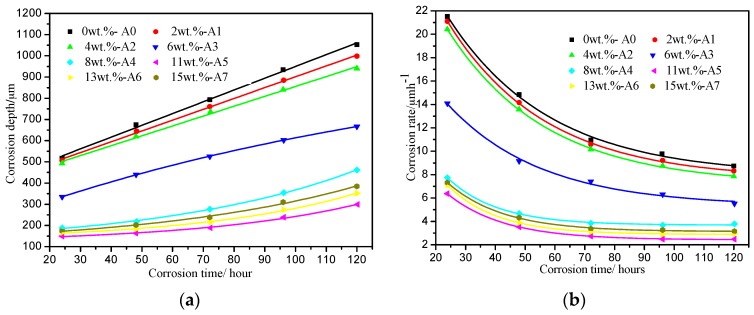
The relationships of corrosion depth (**a**) and corrosion rate (**b**) with corrosion time.

The corrosion depth and corrosion rate curves of Fe-3.5B-*x*W alloys are shown in Figure 4 as functions of corrosion time. The corrosion depth increases with an increase in the corrosion time in all the specimens. The samples containing tungsten exhibit better corrosion resistance to liquid zinc when compared with the Fe-3.5B binary alloy. For the same corrosion time, as the addition of W increases, the corrosion depth decreases, and this situation exists when the W content is less than 11 wt %; however, the corrosion depth increases as W exceeds 11 wt %. The A5 alloy containing 11 wt % W has the least corrosion depth and shows the best corrosion resistance in liquid zinc. A further observation reveals that all the samples have high corrosion rates during the early stages of corrosion. After 3 days, the corrosion process in all the samples slows down.

**Figure 5:**
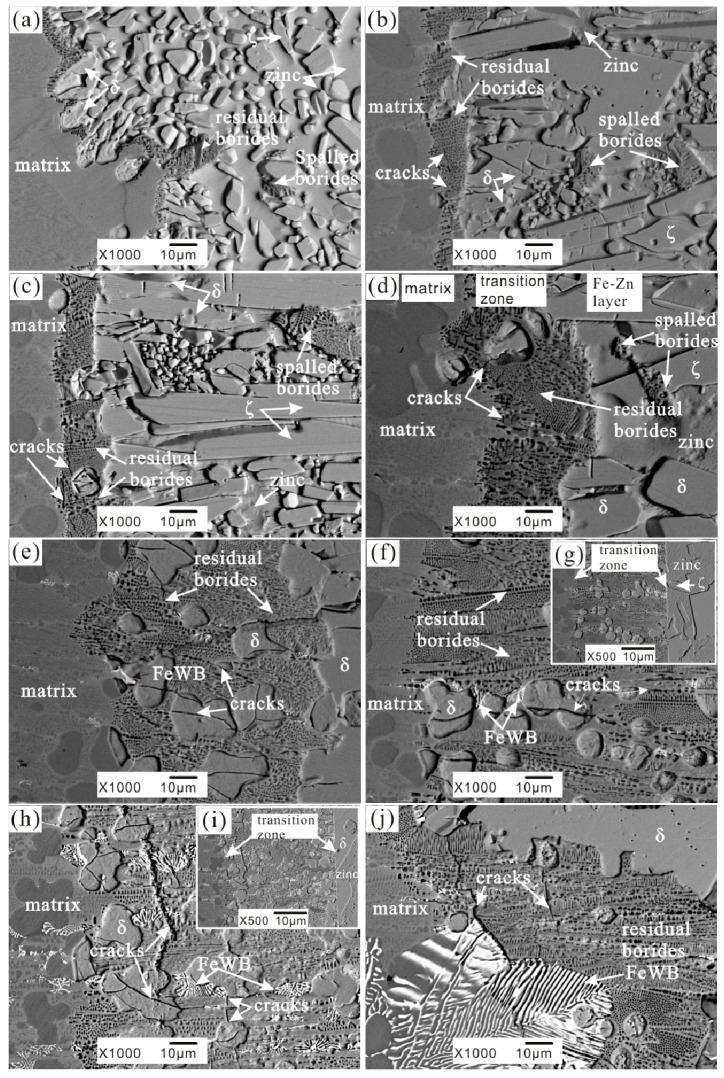
The morphology of corrosion layer in Fe-3.5B alloys containing different amounts of tungsten immersed in liquid zinc for 72 h at 520 °C (**a**) A0-0 wt % W (**b**) A1-2 wt % W (**c**) A2-4 wt % W (**d**) A3-6 wt % W (**e**) A4-8 wt % W (**f**) A5-11 wt % W (**g**) the transition zone of A5-11 wt % W (**h**) A6-13 wt % W (**i**) the transition zone of A6-13 wt % W (**j**) A7-15 wt % W.

Figure 5 shows the cross-section morphologies of the corrosion interface of the samples dipped into liquid zinc for 72 h. For the pristine Fe-3.5B alloy with no tungsten, the interface is highly irregular. There are many dispersed Fe-Zn compound particles, some spalled borides, and a few residual boride skeletons attached to the matrix. With the addition of W, it can be seen clearly that the cross sections of the corroded samples display three different regions in the backscattered electron image (BSE)—the un-corroded matrix, a transition region, and a Fe-Zn compound layer. The transition region is comprised of the skeleton of borides and Fe-Zn compounds. It is deduced that the corrosion resistance of borides is better than that of the α-(Fe, W). The morphology of the Fe-Zn compound layer (the bright region in the BSE image) consists of massive metallic particles separated by channels filled with liquid zinc.

With the increase of the W addition in the Fe-3.5B alloy, the boride skeleton in the transition layer becomes thicker. A thick and complete boride skeleton is helpful in improving the corrosion resistance of the Fe-B alloy because it can act as a diffusion barrier to the Fe-Zn reaction. From Figure 5h,j we can see that when the W content is more than 13 wt %, several cracks appear across the (Fe, W)_2_B + (Fe, W)_3_B net and the white FeWB phase; ultimately, these cracks result in the spalling of the borides along the fracture.

**Figure 6:**
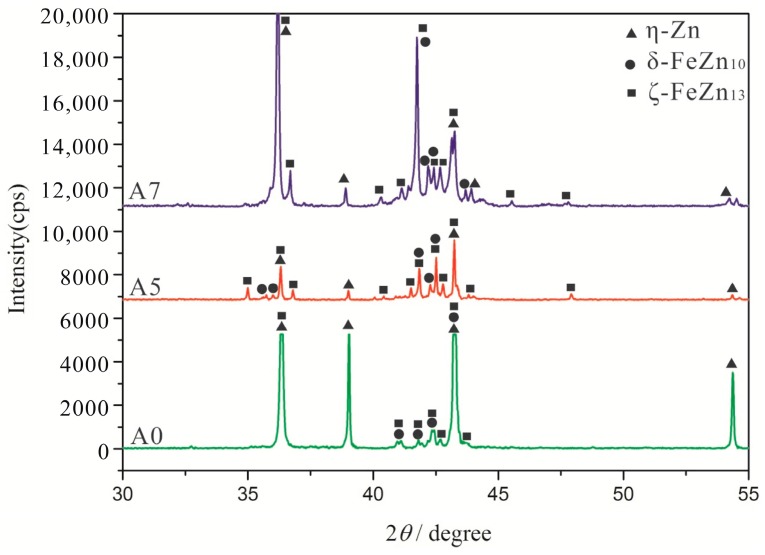
X-ray diffraction patterns of corrosion products of the A0, A5 and A7 alloys at 520 °C.

Figure 6 depicts the XRD patterns of the corrosion products of the A0, A5 and A7 alloys. The corrosion layer is determined to be composed of δ-FeZn_10_ (JCPDS 45-1184), ζ-FeZn_13_ (JCPDS 65-1238), and η-Zn (JCPDS 65-3358).

### 3.3. Element Distribution in the Corrosion Layer

**Figure 7:**
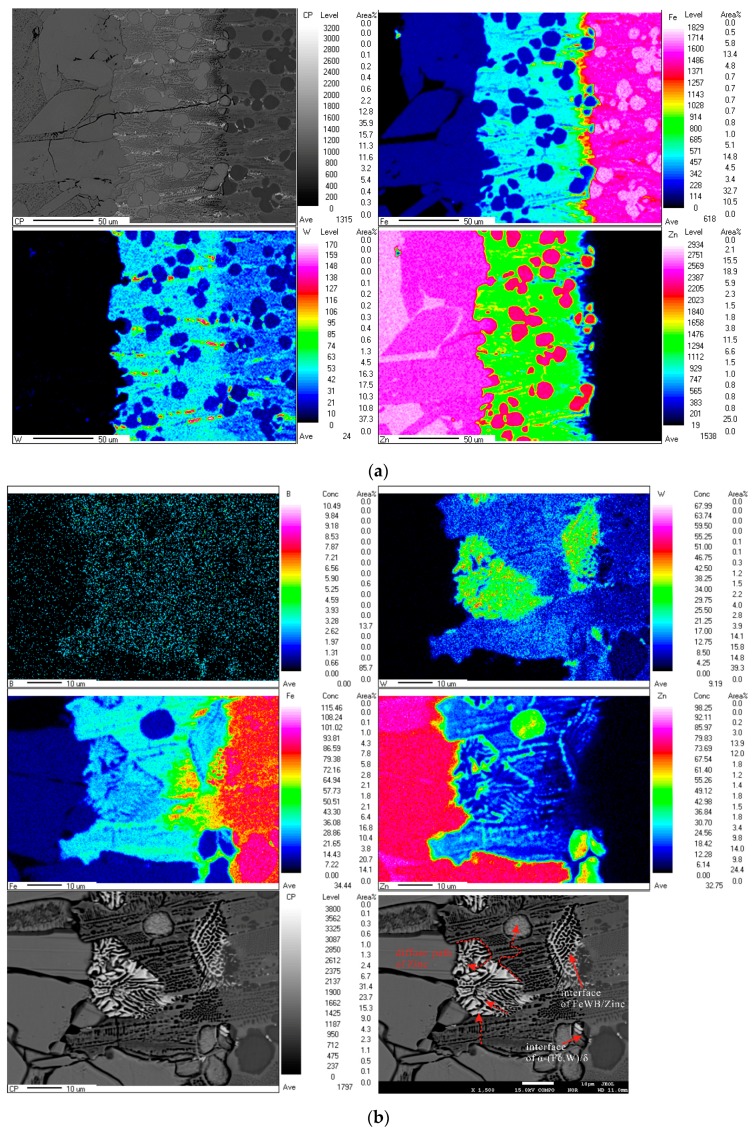
Element EPMA mapping of corrosion layer for alloy A5 (**a**) and A7 (**b**).

In order to understand the corrosion behavior, element distribution in the corrosion layers of the alloys A5 and A7 were investigated by EPMA. The surface scanning mapping of EPMA illustrates the distribution of Fe, W, Zn, and B in different colors. The colored scale bars at the right show the relative concentrations of different elements. The EPMA mapping result of A5 alloy (Figure 7a) shows that Fe content significantly decreases while Zn increases from the matrix to the liquid zinc layer. It reveals that the borides in the transition region have a little dissolved zinc, while the content of zinc in the α-Fe matrix is very high. It implies that the α-(Fe, W) matrix can be corroded easily. From the Zn distribution, we can see that there are two types of Fe-Zn compounds. The first type (in pink color), with higher Zn content is located outside, whereas the second type with a relatively low Zn content (in red color) is encircled by a boride net. They are the δ_k_ and δ_p_ phases, respectively. With regard to the distribution of tungsten, its content in the transition region is higher than in both the Fe-Zn layer and the uncorroded areas. The explanation for this phenomenon is as follows—the diffusion of W atom into liquid zinc is difficult due to its large atomic radius and low solubility in zinc. Meanwhile Fe is able to dissolve in zinc and form Fe-Zn metallic particles. The combined effects of Fe, Zn, and W inter-diffusion are responsible for the high W content in the transition layer.

Figure 7b shows the EPMA mapping results of alloy A7 (with flowerlike white ternary borides). FeWB and reticular borides both have excellent corrosion resistance to liquid zinc, but in the microstructure of A7 we can see distinct micro-cracks. In the FeWB boride areas, the content of both Fe and W increase. The mapping result for Zn shows its rapid diffusion in the corrosion layer. The Zn atom prefers to diffuse along the boundary of the FeWB boride and the fast diffusion path coincides with the crack. The micro-cracks initiated from the interface of FeWB and reticular borides will cause the boride skeleton to spall off eventually.

## 4. Discussion

### 4.1. Effects of W Addition on the Microstructure of the Fe-3.5B Alloy

**Figure 8:**
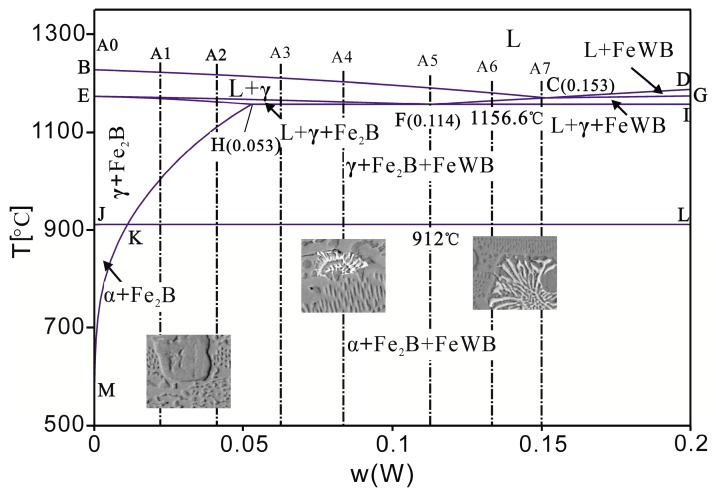
The calculated vertical section of the Fe-B-W ternary system with boron fixed at 3.5 wt % compared with the microstructures of typical alloys.

In order to understand the effect of W on the microstructure of the Fe-B alloy, the phase relationship of the Fe-B-W ternary system is extrapolated using the CALPHAD method, based on the information available on the Fe-B, B-W, and Fe-W binary systems [21,22,23]. The calculated vertical section of the ternary system with B fixed at 3.5 wt % is shown in Figure 8. From the vertical section, we can see there is a L → γ + Fe_2_B + FeWB eutectic reaction at 1156.6 °C, the eutectic composition of the liquid phase is 11.4 wt %. For alloy A1-A2, when W addition is less than 5.3 wt %, the crystallization reaction is L → γ and L → γ + Fe_2_B along the BC and EF lines during the casting process. The solidification process for A3-A4 alloy is L → L + γ → L + γ + Fe_2_B → γ + Fe_2_B + FeWB, and the small FeWB domains are few in number. In the A5 alloy which is closest to the eutectic composition, the L → L + γ → γ + Fe_2_B + FeWB solidification process takes place. For the A6 alloy, the solidification process is L → L + γ → L + γ + FeWB → γ + Fe_2_B + FeWB, and some γ + FeWB flowerlike microstructures are generated in the system. For the A7 alloy, with the increasing W addition, the volume fraction and the grain size of the flowerlike microstructures increase. With the solid content increasing during the casting process, the diffusion of W and B becomes even harder, and the grain sizes of the Fe_2_B borides become much smaller. The experimental results of the effects of W on the as-cast microstructure of the Fe-B alloys and the calculated phase diagram are in good agreement.

**Figure 9:**
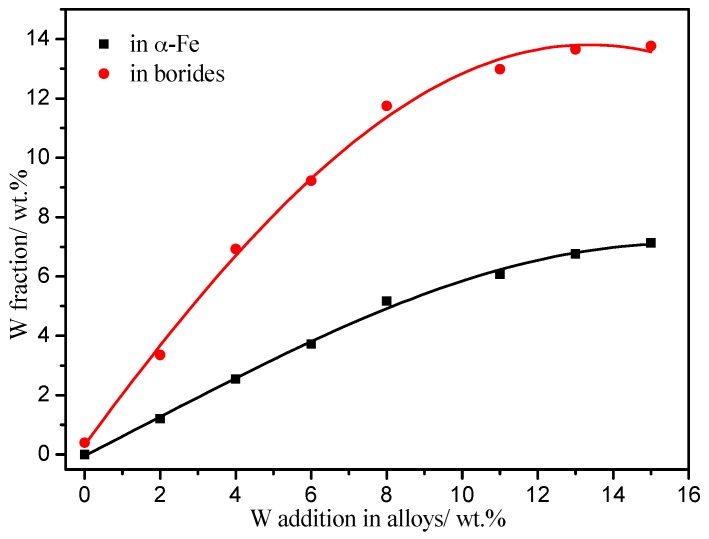
Relationship of the W content in the Fe_2_B and α-Fe phases with the addition of W in the Fe-3.5B cast alloys.

Figure 9 shows the relationship of the W content in the Fe_2_B and α-Fe phases with the addition of W in the Fe-3.5B cast alloys. With increasing W addition, the W fractions in both α-Fe and Fe_2_B increase. The W fractions in Fe_2_B are much higher than that in the α-Fe phase at any given value of W addition. The maximum W fraction in α-Fe is about 7 wt %. With the addition of W in α-Fe, the corrosion resistance of the matrix is improved due to the lower atomic mobility of W. The maximum W fraction in Fe_2_B is about 14 wt % for the A6 alloy. As in Huang’s research, with the addition of tungsten the fracture toughness and the hardness of Fe_2_B were improved [19].

According to Leithe and Okamoto’s works, a large supercooling in the casting process and the addition of tungsten are beneficial to the formation of a metastable Fe_3_B phase [24,25]. Our research demonstrates that conclusion. As the diffusion of W and B becomes difficult during the cooling process, the W element is enriched in the solid/liquid interface. The (Fe, W)_3_B domains nucleate preferentially at the grain boundary, and the growth of α-(Fe, W) is prevented, resulting in as-cast microstructures with a refined α-(Fe, W) wrapped by borides.

### 4.2. Effect of W Addition on the Corrosion Resistance of the Fe-3.5B Alloy

According to Ma’s reports [11], the α-Fe matrix may be the first phase to be corroded when the Fe-3.5B alloy is immersed in liquid zinc. The dense continuous network of the Fe_2_B phase can hinder the Fe/Zn interface reaction, but the spalling of Fe_2_B reduces the potency of the hindrance effect [26,27]. Therefore, the core factors affecting the corrosion resistance of the Fe-3.5B-*x*W alloy in liquid zinc are the corrosion assistance of the α-Fe matrix, and the stability of the borides. With the addition of W, the matrix of the supersaturated α-(Fe, W) solid solution in the Fe-3.5B-*x*W alloys can slow down the reaction between the substrate and liquid zinc. In addition, the compact δ phase with its relatively low Zn content, when formed on the transition zone, can also hinder the Fe/Zn interface reaction. Thus the corrosion resistance of the matrix is improved.

**Figure 10:**
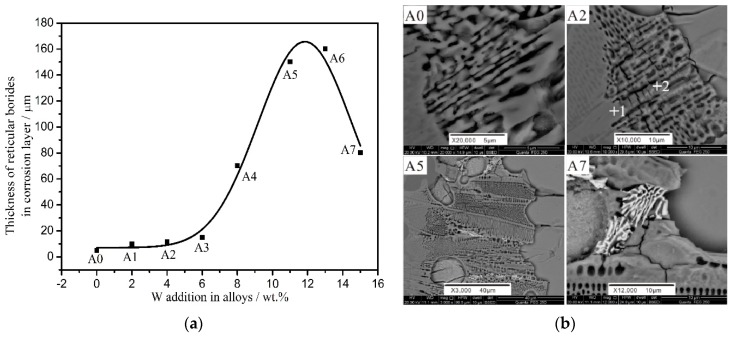
(**a**) The relationship of the average thickness of reticular borides of all the alloys and (**b**) the SEM results of reticular borides of A0, A2, A5 and A7 alloys in the transition zone after 3 days of immersion in liquid zinc with the addition of W.

Regarding the stability of the borides, if the transition region is thick, the diffusion of the zinc atom becomes more difficult; therefore, a thick and complete boride layer is important for corrosion resistance. The average thicknesses of the reticular borides in the transition zone of all the alloys were measured after 3 days of immersion in liquid zinc (Figure 10). With increasing W addition, the thickness of the transition region initially increases and experiences a reduction in the later stages. The A5 alloy possesses the thickest transition region among all the samples. From the SEM results in Figure 10, (A0, A2, A5 and A7), we can see that when the W content is less than 11 wt %, there is an obvious spheroidization of the Fe_2_B phase, and some transverse cracks are initiated from the oriented (Fe, W)_3_B. If the W content is more than 13 wt %, the big flowerlike FeWB microstructures and the cracks across the borides destroy the completeness of the net structure. The spheroidization or flowerlike morphology and the cracks weaken the stability of the borides, therefore, the stability of borides in Fe-3.5B-11W alloy is the best.

As for the borides ((Fe, W)_2_B, (Fe, W)_3_B, and FeWB), from the WDX analysis (Table 3) of the transition region in alloy A2, it can be inferred that both (Fe, W)_2_B and (Fe, W)_3_B undergo the transformation from (Fe, W)_2_B or (Fe, W)_3_B to (Fe, W)B; the corrosion products of this reaction are FeBZn_*x*_. Studying the corrosion mechanism of FeWB in liquid zinc is beyond the scope of this paper, and the essential process needs to be investigated in future. Nevertheless, it is understood the micro-cracks initiated from the interface of the borides lead to their spalling [26,27,28].

### 4.3. Corrosion Mechanism of Fe-3.5B-xW Alloys

**Figure 11:**
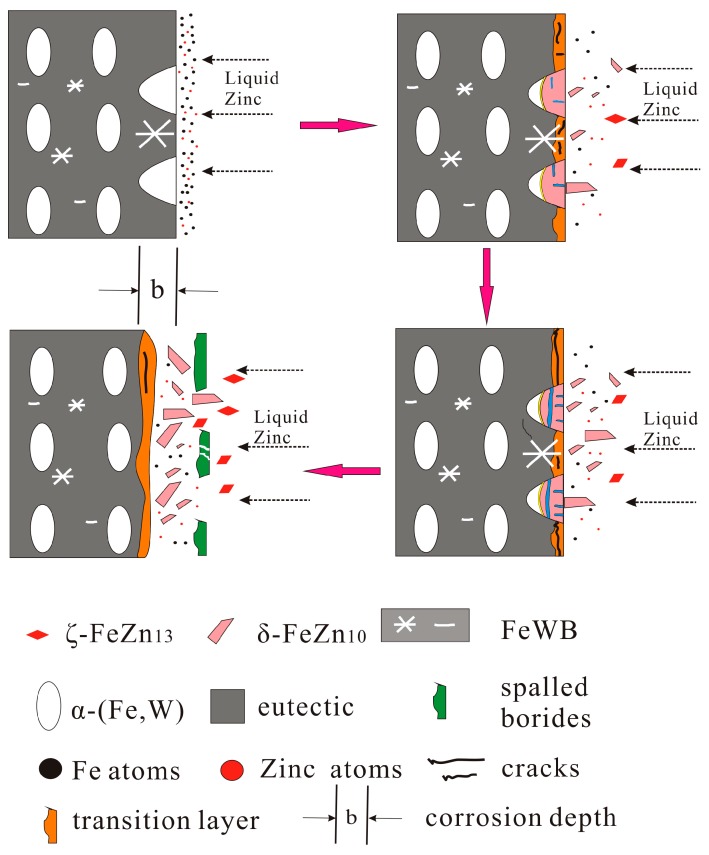
Schematic of corrosion mechanism of cast alloys dipped into liquid zinc.

The whole corrosion behavior of Fe-3.5B-*x*W alloys in liquid zinc is represented schematically in Figure 11, it involves four physical and physicochemical processes.

(1) Corrosion of the α-(Fe, W) matrix. As the metal matrix comes into contact with liquid zinc, the α-(Fe, W) phase is the first victim of corrosion. A succession of Γ, Γ_1_, δ, ζ, and η-Zn compounds are generated. In this paper, the existences of δ, ζ, and η-Zn phases are confirmed by XRD; however, the Γ phase is too thin to be detected.
(2) Formation of cracks along the reticular borides. Numerous Fe-Zn compounds and the refined reticular borides can act as a barrier and impede the diffusion of liquid zinc. Hence, it is difficult for zinc atoms to find the defects through which they can diffuse in the completeness of the boride net structure. Due to the different coefficients of thermal expansion of the Fe-Zn compounds a lot of micro-cracks are initiated in the reticular borides and the diffusion channels for zinc atoms are formed.
(3) Formation of the transition region. The Zn atoms diffuse along the cracks to the α-(Fe, W) wrapped by the reticular borides, and the δ phase is generated. As the content of Zn is not high, the borides still maintain a relatively intact reticular structure. This leads to a transition region consisting of the uncorroded boride and δ compounds. Meanwhile, the cracks become very obvious.
(4) Spalling of the reticular borides. The cracks are further propagated along the borides and the corrosion products are transported by thermo-stress and the flow of liquid zinc. Eventually, the borides are spalled off.

The effects of W addition on the corrosion resistance of Fe-B alloys in liquid zinc can be summarized into three components. First, as the W atoms enter into the α-Fe phase, the corrosion resistance of the matrix is improved, and the corrosion products with their compact microstructure can also delay the diffusion of zinc. Secondly, the refined borides with their reticular structure can resist the diffusion of zinc atoms, resulting in a low corrosion rate. Third, the borides containing W do enhance the stability of the skeleton structure dipped in liquid zinc, for a long period of time. Therefore, the Fe-3.5B alloy with 11 wt % tungsten has the best corrosion resistance.

## 5. Conclusions

The aim of this study is to investigate the effect of W addition on the microstructure and the corrosion resistance of a Fe-B alloy in liquid zinc. The following conclusions are obtained from the results:

(1) The morphology of the borides in the Fe-3.5B-*x*W alloys is different, several types exist, such as the reticular borides (Fe, W)_2_B, the rod borides (Fe, W)_3_B, and the white borides FeWB with their flower-like shape. Furthermore, the network structure of the eutectic borides is refined significantly by W inclusion.
(2) The alloy with a 11 wt % W addition exhibits the best corrosion resistance to liquid zinc at 520 °C. The corrosion of the α-(Fe, W) phase occurs preferentially. As W addition increases, the corrosion resistance of the α-(Fe, W) phase is improved. The W addition can also improve the stability of the borides.
(3) Micro-cracks are initiated along the boride grain boundaries, and result in the fracture-spalling of the borides.

## Figures and Tables

**Table 1:** The nominal chemical composition of Fe-3.5B alloys (wt %).

Samples	B	C	Si	W	Fe
A0	3.50	0.10–0.15	0.43–0.51	0	Balance
A1	3.50	0.10–0.15	0.43–0.51	2	Balance
A2	3.50	0.10–0.15	0.43–0.51	4	Balance
A3	3.50	0.10–0.15	0.43–0.51	6	Balance
A4	3.50	0.10–0.15	0.43–0.51	8	Balance
A5	3.50	0.10–0.15	0.43–0.51	11	Balance
A6	3.50	0.10–0.15	0.43–0.51	13	Balance
A7	3.50	0.10–0.15	0.43–0.51	15	Balance

**Table 2:** Composition of phases in typical cast alloys confirmed by WDX (at %).

Sample	Phase	Fe	W	B	Ratio of Fe and B
A0	α-Fe	100.00	-	-	-
	Fe_2_B	67.90	-	32.10	2:1
A7	α-(Fe, W)	98.97	1.03	-	-
	(Fe, W)_3_B	75.56	3.11	21.33	3:1
	FeWB	34.05	32.28	33.67	1:1

**Table 3:** Chemical composition of the transition region in alloy A2 analyzed by EPMA in Figure 10.

Number	Fe		W		B		Zn		Phase
	wt %	at %	wt %	at %	wt %	at %	wt %	at %	
1	81.97	73.93	11.35	3.11	4.58	21.34	2.10	1.62	(Fe, W)_3_B
2	42.19	37.93	32.48	8.87	8.70	40.42	16.63	12.77	FeBZn*_x_*
